# The Effect of Various Doses of Phenylalanine Supplementation on Blood Phenylalanine and Tyrosine Concentrations in Tyrosinemia Type 1 Patients

**DOI:** 10.3390/nu11112816

**Published:** 2019-11-18

**Authors:** Willem G. van Ginkel, Hannah E. van Reemst, Nienke S. Kienstra, Anne Daly, Iris L. Rodenburg, Anita MacDonald, Johannes G.M. Burgerhof, Pim de Blaauw, Jennifer van de Krogt, Saikat Santra, M. Rebecca Heiner-Fokkema, Francjan J. van Spronsen

**Affiliations:** 1 Department of Metabolic Diseases, Beatrix Children’s Hospital, University Medical Center Groningen, University of Groningen, 9713 GZ Groningen, The Netherlands; w.g.van.ginkel@umcg.nl (W.G.v.G.); h.e.van.reemst@student.rug.nl (H.E.v.R.); n.s.kienstra@student.rug.nl (N.S.K.); 2 Department of Metabolic Diseases, Birmingham Children’s Hospital, Birmingham B4 6NH, UK; a.daly3@nhs.net (A.D.); anita.macdonald@nhs.net (A.M.); s.santra@nhs.net (S.S.); 3 Department of Dietetics, University Medical Center Groningen, University of Groningen, 9713 GZ Groningen, The Netherlands; i.l.rodenburg@umcg.nl; 4 Department of Epidemiology, University Medical Center Groningen, University of Groningen, 9713 GZ Groningen, The Netherlands; j.g.m.burgerhof@umcg.nl; 5 Department of Laboratory Medicine, University Medical Center Groningen, University of Groningen, 9713 GZ Groningen, The Netherlands; p.de.blaauw@umcg.nl (P.d.B.); j.van.der.krogt@umcg.nl (J.v.d.K.); m.r.heiner@umcg.nl (M.R.H.-F.)

**Keywords:** tyrosinemia type 1, phenylalanine, supplementation, tyrosine

## Abstract

Tyrosinemia type 1 (TT1) treatment with 2-(2-nitro-4-trifluormethyl-benzyl)-1,3-cyclohexanedione (NTBC) and a phenylalanine-tyrosine restricted diet is associated with low phenylalanine concentrations. Phenylalanine supplementation is prescribed without comprehensive consideration about its effect on metabolic control. We investigated the effect of phenylalanine supplementation on bloodspot phenylalanine, tyrosine, NTBC and succinylacetone. Eleven TT1 patients received 0, 20 and 40 mg/kg/day phenylalanine supplementation with the phenylalanine-tyrosine free L-amino acid supplements. Bloodspots were collected before breakfast, midday and evening meal. Differences between study periods, sample times and days within a study period were studied using (generalized) linear mixed model analyses. Twenty and 40 mg/kg/day phenylalanine supplementation prevented daytime phenylalanine decreases (*p* = 0.05) and most low phenylalanine concentrations, while tyrosine concentrations increased (*p* < 0.001). Furthermore, NTBC and succinylacetone concentrations did not differ between study periods. To conclude, 20 mg/kg/day phenylalanine supplementation can prevent most low phenylalanine concentrations without increasing tyrosine to concentrations above the target range or influencing NTBC and succinylacetone concentrations, while 40 mg/kg/day increased tyrosine concentrations to values above the targeted range. Additionally, this study showed that the effect of phenylalanine supplementation, and a possible phenylalanine deficiency, should be assessed using pre-midday meal blood samples that could be combined with an overnight fasted sample when in doubt.

## 1. Introduction

Tyrosinemia type 1 (TT1; McKusick 276700) is a rare, autosomal recessive disorder of tyrosine metabolism caused by a deficiency of fumarylacetoacetate hydrolase, the last enzyme of the tyrosine degradation pathway (incidence 1:100.000). Without treatment, this deficiency results in the accumulation of toxic metabolites prior to the enzyme defect, such as maleylacetoacetate, fumarylacetoacetate, succinylacetoacetate and succinylacetone (SA), causing acute liver failure, hepatocellular carcinoma (HCC), renal tubulopathy, and porphyria-like-syndrome with neuropathy [1].

Before the introduction of 2-(2-nitro-4-trifluormethyl-benzyl)-1,3-cyclohexanedione (NTBC), when patients were treated with a diet only, the outcome of TT1 was very poor [2,3]. NTBC, however, prevents the accumulation of toxic metabolites by blocking 4-OH-phenylpyruvate dioxygenase, an enzyme proximal from the primary enzyme defect [4]. In this way, NTBC led to the resolution of liver failure and porphyria-like-syndrome, a substantial reduction in HCC and consequently eliminated the need for liver transplantation [5,6,7]. However, due to the metabolic block caused by NTBC, tyrosine levels increased, and so a phenylalanine-tyrosine restricted diet remained necessary [8].

Combined treatment with NTBC and diet resolved most of the clinical problems. However, during longer term follow-up, it is challenging to maintain both tyrosine and phenylalanine concentrations within target range. As a consequence, low phenylalanine concentrations are reported frequently [4,9,10,11,12,13]. It has been shown that phenylalanine concentrations tend to show a diurnal variation with lowest concentrations during the afternoon [13]. Low phenylalanine concentrations are associated with poor growth, eczema-like skin eruptions and developmental delay in infants [11], and behavioral problems later in life [14]. To increase phenylalanine concentrations in TT1 patients, phenylalanine supplementation may be prescribed despite the likely conversion of some phenylalanine to tyrosine [9,11,12]. As phenylalanine supplementation increases the flux through the phenylalanine-tyrosine catabolic pathway, phenylalanine supplementation might not only increase tyrosine concentrations but, at least theoretically, increase blood SA concentrations as well. As blood NTBC concentrations may also influence blood SA and consequently also tyrosine and phenylalanine concentrations, NTBC concentrations are needed to study differences in SA, tyrosine and phenylalanine concentrations. 

Therefore, this study aimed to investigate the biochemical effect of different amounts of phenylalanine supplementation on (1) blood phenylalanine and tyrosine concentrations and (2) blood SA and NTBC concentrations. 

## 2. Materials and Methods 

### 2.1. Subjects

In total, 11 TT1 patients (7 males, 4 females; mean age 14.0 years; range 6.9–27.0 years) were studied. Five patients (subjects 1–5) were diagnosed and treated in the University Medical Center Groningen (UMCG) (The Netherlands) and six patients (subject 6–11) in the Birmingham’s Children’s hospital (UK). All patients received NTBC and a protein restricted diet with phenylalanine and tyrosine free L-amino acids supplements. The dietary prescription remained unchanged during the study. All subjects maintained their regular NTBC dose. If phenylalanine supplementation was used as part of their standard treatment (*N* = 4), the supplementation was stopped one week before the study start. 

The study was approved by the medical ethical committee of the UMCG in The Netherlands and by the UK South Birmingham ethical committee. All TT1 patients and/or their caregivers gave written informed consent for this study. 

### 2.2. Study Design

This study consisted of three different study periods in which three blood spots a day were taken at home by the parents/guardians of the patients or by the patients themselves. The blood spots were taken before breakfast, lunch (also called midday meal) and dinner (also called evening meal). The first study period consisted of two days in which no phenylalanine supplementation was given. During the second study period, 20 mg/kg/day phenylalanine supplementation was given for five consecutive days. During the third study period, 40 mg/kg/day phenylalanine supplementation was given for five consecutive days. This third study period started after a wash-out period of seven days in which patients did not take any phenylalanine supplementation. The phenylalanine supplementation was divided into 3 doses/day, taken during meals together with the phenylalanine and tyrosine free L-amino acid supplements. During the periods with phenylalanine supplementation, bloodspots were taken during the last 4 days of the study period (Table 1).

### 2.3. Phenylalanine Capsules

For both centers, capsules containing 50 mg and 100 mg of L-phenylalanine were prepared by the pharmacy in the University Medical Center Groningen.

### 2.4. Blood Spot Sampling

Phenylalanine, tyrosine, NTBC and SA concentrations were analyzed by blood sampling via collecting blood spots on blood spot cards. All blood spots were collected at home through finger punctures on blood spot cards made of filter paper (Grade FN 179 g/m2 Satorius, Göttingen, Germany). Two bloodspot cards were used for each sample collected. The first bloodspot card measured phenylalanine and tyrosine (and NTBC in patients 6–11) and was stored at room temperature until analyses. The second bloodspot card measured SA (and NTBC in patients 1–5). This blood spot card was dried for at least 3 h at room temperature and afterwards stored at −20° Celsius in seal bags with a silica sachet (at home and in laboratory after transport using dry ice) until analyses. Blood spot phenylalanine, tyrosine, NTBC and SA concentrations were analyzed using high-performance liquid chromatography coupled to tandem mass spectrometry. The limit of quantification for SA was 0.1 µmol/L. All bloodspots were analyzed at the laboratory of the UMCG.

### 2.5. Statistical Analysis

Differences in blood phenylalanine, tyrosine and NTBC concentrations between the different study periods (0, 20, 40 mg/kg/day phenylalanine supplementation), days within a study period, and sample times (diurnal variation) were studied using linear mixed effects models. NTBC concentrations were analyzed after logarithmic transformation. Results on blood SA concentrations were divided into SA < 0.1 µmol/L and SA ≥ 0.1 µmol/L. Differences in SA concentrations between the different study periods were studied using generalized linear mixed model analyses. Additionally, generalized linear mixed model analyses were undertaken to study the correlation between blood SA concentrations and blood tyrosine and NTBC concentrations. Statistical analyses were conducted with the statistical program SPSS 23 (IBM, Chicago, IL, USA). All tests were performed two-sided and a *p*-value of 0.05 was considered statistically significant. Graphs were made using Graphpad Prism 7.

## 3. Results

### 3.1. Patient Characteristics 

Eleven patients diagnosed with TT1 were recruited from the UMCG, the Netherlands and Birmingham Children’s Hospital, UK. Patient characteristics are shown in Table 2. In five patients, the total daily dose of NTBC was divided into two doses (patients 1–5), six patients were given a single daily dose of NTBC in the morning (patients 6–11). All patients maintained their individually tailored phenylalanine and tyrosine restricted diet with phenylalanine and tyrosine free supplementary L-amino acids. During the study, patients were asked to document possible symptoms associated with phenylalanine supplementation. No patients reported any new or exacerbation of pre-existing clinical problems possibly associated with the additional phenylalanine.

### 3.2. Blood Phenylalanine and Tyrosine Concentrations

**Figure 1:**
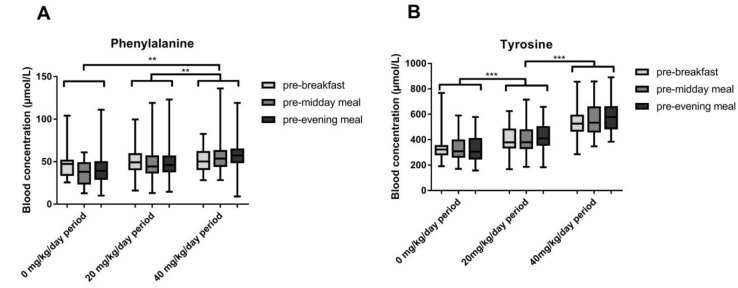
Mean blood phenylalanine (**A**) and tyrosine (**B**) concentrations during the different study periods presented as min–max whiskers. Figure 1A shows the difference in diurnal variation of blood phenylalanine concentrations between the different study periods (*p* = 0.05) and higher blood phenylalanine concentrations when 40 mg/kg/day phenylalanine supplementation is given compared to both the study period with none (*p* = 0.001) and 20 mg/kg/day phenylalanine supplementation (*p* = 0.005). Figure 1B shows an increase in tyrosine concentrations when both 20 mg/kg/day and 40 mg/kg/day phenylalanine supplementation is given (both *p <* 0.001). **p < 0.01, ***p < 0.001.

Figure 1A shows mean blood phenylalanine concentrations at the three different sample times during all study periods. A linear mixed model analysis was done with blood phenylalanine concentrations as the dependent variable with study period (0, 20 and 40 mg/kg/day phenylalanine supplementation) and sampling time (breakfast, midday meal, evening meal) as factors. This analysis showed a significant interaction between the study period and sampling times, indicating differences in diurnal variation between the different study periods (*p* = 0.05). Without any supplementation, mean phenylalanine concentrations decreased from 50 ± 21 µmol/L at breakfast to 37 ± 14 µmol/L before the midday meal (Table 3). During this study period without phenylalanine supplementation, nine out of 11 patients had a blood phenylalanine concentration <30 µmol/L on at least one occasion. With 20 mg/kg/day phenylalanine supplementation, the decrease in phenylalanine was less pronounced (from 51 ± 18 µmol/L to 47 ± 19 µmol/L). Additionally, only four patients had blood phenylalanine concentrations <30 µmol/L when 20 mg/kg/day phenylalanine supplementation was prescribed. When receiving 40 mg/kg/day of phenylalanine supplementation, there was no decrease in blood phenylalanine concentration in the course of the day when compared to morning blood phenylalanine concentrations, with mean blood phenylalanine concentrations of 52 ± 14 µmol/L before breakfast and 56 ± 20 µmol/L before the midday meal. During this study period, only two patients had blood phenylalanine concentrations <30 µmol/L. One patient seemed not to respond to both doses of phenylalanine supplementation, with low phenylalanine concentrations in each study period. The other patient showed two consecutive low phenylalanine concentrations while receiving 40 mg/kg/day phenylalanine supplementation, whereas all other phenylalanine concentrations during this study period were within normal range.

The same linear mixed model analysis, with phenylalanine concentrations as the dependent variable, showed that, apart from the significant interaction mentioned in the previous paragraph, phenylalanine concentrations were also significantly different between study periods (*p* < 0.001). Blood phenylalanine concentrations were significantly lower in study periods one and two when compared to study period three (*p* = 0.001 and *p* = 0.005 respectively). Mean blood phenylalanine concentrations during the whole study period increased from 43 ± 21 µmol/L without supplementation to 49 ± 20 and 55 ± 18 µmol/L with 20 and 40 mg/kg/day phenylalanine supplementation, respectively. 

Figure 1B shows mean blood tyrosine concentrations during the study at the different sample times. Linear mixed model analysis with blood tyrosine concentrations as the dependent variable with study period (0, 20 and 40 mg/kg/day phenylalanine supplementation) and sampling time (breakfast, midday meal, evening meal) as factors showed that blood tyrosine concentrations were significantly different between the different study periods (*p* < 0.001). Without phenylalanine supplementation, mean blood tyrosine concentrations were 339 ± 117µmol/L, while blood tyrosine concentrations increased to 409 ± 112 µmol/L and 558 ± 127 µmol/L with 20 and 40 mg/kg/day phenylalanine supplementation, respectively. Without phenylalanine supplementation, blood tyrosine concentrations >400 µmol/L were found at least once during the study period in 4/11 patients (24% of all samples). When receiving 20 or 40 mg/kg phenylalanine supplementation, tyrosine concentrations >400µmol/L were observed in 9/11 (47% of all samples) and 11/11 patients (92% of all samples), respectively. Blood tyrosine concentrations did not differ significantly between the different sample times (*p* = 0.052). 

**Figure 2:**
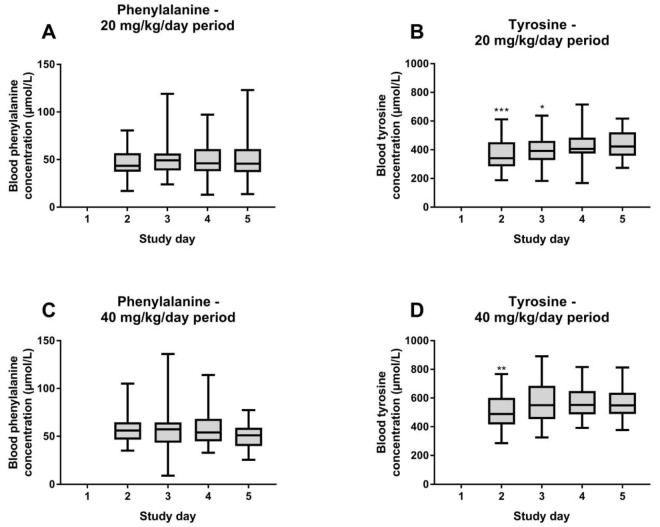
Day-to-day variation of blood phenylalanine and tyrosine concentrations while receiving 20 (**A**,**B**) and 40 mg/kg/day (**C**,**D**) phenylalanine supplementation, presented as min–max whiskers. In both periods, no bloodspots were taken at the first day of the study period followed by three bloodspots a day during four consecutive days. Phenylalanine and tyrosine concentrations at each day were compared to the phenylalanine and tyrosine concentrations at the last study day of that study period. * *p* < 0.05, ** *p* < 0.01, *** *p* < 0.001.

To assess day-to-day variation in both study periods with phenylalanine supplementation, linear mixed model analyses were performed with blood phenylalanine or tyrosine concentrations as dependent variable and study day as factor. Figure 2 shows this day-to-day variation of phenylalanine and tyrosine concentrations while receiving different amounts of phenylalanine supplementation. Blood phenylalanine concentrations did not differ from day-to-day while receiving 20 or 40 mg/kg/day phenylalanine supplementation. However, while receiving 20 mg/kg/day phenylalanine supplementation, blood tyrosine concentrations were significantly lower at the second (*p* < 0.001) and third (*p* = 0.016) day of the study period when compared to the last day of that particular study period. While receiving 40 mg/kg/day phenylalanine supplementation, blood tyrosine concentrations were only significantly lower at the second day of the study period (*p* = 0.002), when compared to the last day of this study period.

### 3.3. Blood NTBC and SA Concentrations

**Figure 3:**
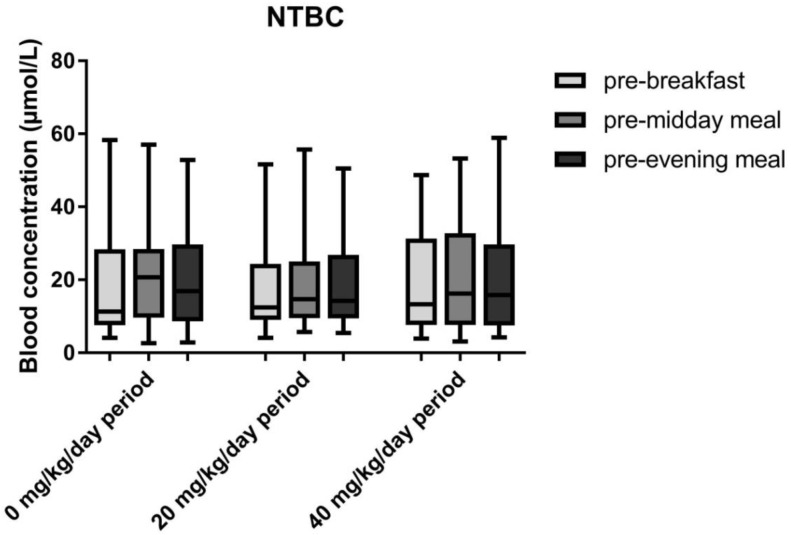
Mean blood NTBC concentrations during the different study periods presented as min–max whiskers. Blood NTBC concentrations showed diurnal variation during all study periods (*p* = 0.021), with lowest concentrations pre-breakfast and highest concentrations pre-midday meal. No differences in blood NTBC concentrations between the study periods (0, 20 and 40 mg/kg/day phenylalanine) were found.

Figure 3 shows mean NTBC concentrations during the study. Linear mixed model analysis with blood NTBC concentrations as the dependent variable with study period (0, 20 and 40 mg/kg/day phenylalanine supplementation) and sampling time as factors (breakfast, midday meal, evening meal) showed no significant difference in NTBC concentrations between the different study periods. Median NTBC concentrations were 15 (IQR: 9–29), 15 (IQR: 9–25), 14 (IQR: 8–31) µmol/L when receiving 0, 20 and 40 mg/kg/day phenylalanine supplementation, respectively. NTBC concentrations were, however significantly different at the different sample times (*p* = 0.021), with lowest concentrations before breakfast and highest concentrations before the midday meal.

Table 4 shows the occurrence of quantitatively detectable SA during the study period. SA analyses could only be performed in 192 out of the 330 samples taken during the study (58%) due to variation in bloodspot quality. These 192 samples were distributed among the different study periods as follows: 35/66 samples taken without phenylalanine supplementation, 80/132 samples taken while receiving 20 mg/kg phenylalanine supplementation, 77/132 samples taken while receiving 40 mg/kg phenylalanine supplementation. SA ≥ 0.1 µmol/L was found in 12/35 samples without phenylalanine supplementation, in 41/80 samples taken while receiving 20 mg/kg phenylalanine supplementation and 33/77 samples taken while receiving 40 mg/kg phenylalanine supplementation. When taking within-subject variation into account, generalized linear mixed model analyses showed no significant difference in the occurrence of SA (SA < 0.1µmol/L or SA ≥ 0.1 µmol/L) between the different study periods (*p* = 0.356), nor between sample moments (*p* = 0.915). 

To further study a possible association between the occurrence of SA > 0.1 µmol/L and both blood tyrosine and NTBC concentrations, generalized linear mixed model analyses were performed. These analyses showed that blood tyrosine concentrations were not significantly correlated to the occurrence of SA > 0.1 µmol/L (*p* = 0.134), while blood NTBC concentrations were negatively correlated to the occurrence of SA > 0.1 µmol/L (*p* < 0.001). 

## 4. Discussion

This study investigated the effect of three different doses of phenylalanine supplementation on phenylalanine, tyrosine, NTBC and SA concentrations. The main findings of this study are fourfold. Firstly, supplementation of phenylalanine prevented the decrease in blood phenylalanine concentrations during the day, thereby preventing most blood phenylalanine concentrations below 30 µmol/L. Secondly, phenylalanine supplementation caused blood phenylalanine concentrations to increase slightly, while it induced an increase in blood tyrosine concentrations especially when 40 mg/kg/day phenylalanine supplementation was given. Thirdly, phenylalanine supplementation did not affect blood SA (and NTBC) concentrations. When combined, these findings suggest a possible role for using phenylalanine supplementation in the treatment for TT1. However, as overnight fasted phenylalanine concentrations did not reflect phenylalanine deficiency adequately, a pre-midday meal sample should be taken to detect and study low phenylalanine concentrations. 

Before discussing these results in more detail, some methodological issues are addressed. As TT1 is a very rare inborn error of metabolism (incidence 1:100.000), the study population is relatively small and heterogeneous regarding age and treatment strategy. Unfortunately, additional descriptive information, especially genetic data, was not known for all patients. Therefore, we could not study possible associations between patients’ genetic background and their response to phenylalanine supplementation. During the study, the pre-existing treatment regimen was continued in order to assess the effect of phenylalanine supplementation systematically. In addition, close follow-up during the study period ensured phenylalanine supplementation and bloodspots were taken according to schedule. SA concentrations could not be determined in some samples mostly because there was not enough material or the quality of the bloodspot was inadequate for analyses. However, blood SA concentrations could be analyzed in more than half of the samples and those samples were equally distributed between the different study periods making statistical comparison of blood SA concentrations between different study periods still reliable. 

As high tyrosine concentrations are associated with eye problems in both tyrosinemia type 2 and TT1 [5,15], and a variable expression of neurocognitive problems in tyrosinemia type 2 [16], most dietary recommendations for TT1 advise to aim for tyrosine concentrations < 400 to 600 µmol/L, which are considered to be safe [8,17,18]. To keep tyrosine concentrations within this range, NTBC treatment in TT1 patients is combined with a protein restricted diet with L-amino acid supplements without tyrosine and its precursor phenylalanine [8]. This study substantiates the finding of other studies, that the combined NTBC and dietary treatment regimen without phenylalanine supplementation may result in the risk to have low phenylalanine concentrations especially around midday meal and during the afternoon [9,10,13]. This post-prandial decrease in phenylalanine concentrations most likely reflects a deficiency caused by insufficient intake [19,20,21]. Phenylalanine deficiency, as defined by sustained low phenylalanine concentrations, have been associated with impaired growth, cortical myoclonus, skin problems and neurological deficits in an infant with TT1 that were successfully treated with extra phenylalanine [11]. Additionally, a range of neurocognitive problems have recently been observed in TT1 patients that were found to be possibly related to low phenylalanine concentrations [11,12,22]. Therefore, low phenylalanine concentrations seem to play at least an equally important or maybe even more important role than high tyrosine concentrations.

Both amounts of phenylalanine supplementation prescribed in this study prevented the day time decrease in blood phenylalanine concentrations. Thereby, very low blood phenylalanine concentrations below 30 µmol/L could largely be avoided in most patients. Consequently, mean blood phenylalanine concentrations rose slightly, especially when 40 mg/kg/day phenylalanine supplementation was given. Phenylalanine supplementation did, however, lead to an increase in tyrosine concentrations. When 20 mg/kg/day phenylalanine supplementation was given, blood tyrosine concentrations showed a small but statistically significant increase although concentrations remain around the target range. However, when patients were treated with 40 mg/kg/day phenylalanine, blood tyrosine concentrations rose significantly to concentrations far exceeding the target range, thereby possibly having clinical relevance. Most likely, phenylalanine is especially converted into tyrosine when blood phenylalanine concentrations are restored to normal. This is also apparent in overnight fasted phenylalanine concentrations, which were within normal range and did not differ between study periods. Consequently, pre-midday meal sampling is more adequate in detecting low phenylalanine concentrations and studying the effect of phenylalanine supplementation than overnight fasted samples. This is in contrast to the advice to measure phenylalanine concentrations before breakfast as has been suggested by us. This advice was based on the fact that blood phenylalanine concentrations were most consistent before breakfast and to take a decrease of 20 µmol/L in phenylalanine concentrations into account [13]. However, considering that a post-prandial decrease in essential amino acids specifically reflects a deficiency, the pre-midday meal blood sampling combined with the overnight blood sampling is more adequate [23]. Considering that tyrosine concentrations did not differ significantly during the day, any moment suffices to study the effect of phenylalanine supplementation on tyrosine concentrations. 

To study whether phenylalanine supplementation has a progressive day-to-day effect on both phenylalanine and (especially) tyrosine concentrations, day-to-day variation of phenylalanine and tyrosine was assessed. While no day-to-day variation of blood phenylalanine concentrations was found, a relative small but statistically significant day-to-day variation of tyrosine concentrations was seen during phenylalanine supplementation. Although tyrosine concentrations already increased shortly after the start of phenylalanine supplementation, concentrations at the first day of measurement were still lower when compared to the last day of that particular study period. However, as day-to-day tyrosine concentrations did not differ between the last two to three days of these study periods, a further increase in tyrosine concentrations is, using both doses of phenylalanine supplementation, not anticipated with longer treatment. Based on these results, future studies could take a longer period of about two days after the start of a dietary intervention into account to be as sure as possible that tyrosine concentrations are completely stabilized. 

Based on studies measuring phenylalanine oxidation, the minimal phenylalanine requirement is 9 mg/kg/day in healthy adults when there is excess of tyrosine [24]. Although dependent on the diet, this minimal requirement would correspond to a natural protein intake around 0.2 g/kg/day, which was attained by all study patients. Both doses of phenylalanine supplementation increased the daily phenylalanine intake considerably in all TT1 patients. As a large part of the phenylalanine is most likely being converted into tyrosine, it is remarkable that 20 mg/kg/day phenylalanine supplementation only increased the tyrosine concentrations by a relatively small amount. Therefore, despite an increase in tyrosine intake, it would be useful to study if 20 mg/kg/day phenylalanine supplementation could be translated into a meaningful increase in natural protein intake, as this would be preferable to patients. 

Phenylalanine supplementation increases the flux through the phenylalanine-tyrosine degradation pathway and as NTBC is a competitive inhibitor of the enzyme 4-OH-phenylpyruvate dioxygenase, we studied if SA concentrations change due to phenylalanine supplementation, while also measuring NTBC concentrations. Despite the long-half time of NTBC, NTBC concentrations showed some variation during the day with lowest concentrations in the morning [25]. Unfortunately, differences in blood SA concentrations between both NTBC dosing regimens (once or twice daily) could not be studied as the daily NTBC dose differed as well and both may have resulted in differences in blood NTBC and consequently SA concentrations. Taking into account the limitations mentioned above, NTBC and SA concentrations were not statistically significant between the different study periods. Furthermore, blood tyrosine concentrations did not correlate significantly with SA concentrations. This indicates that despite the increased flux through the phenylalanine catabolic pathway, phenylalanine supplementation did not increase blood SA concentrations with the current NTBC treatment. In fact, considering the clear association between blood NTBC and SA concentrations, maintaining adequate blood NTBC concentrations is much more important to prevent the occurrence of increased SA concentrations. 

## 5. Conclusions

The findings in this study show that: (1) 20 mg/kg/day phenylalanine supplementation could prevent most of the low blood phenylalanine concentrations observed during the day, that often occur when no additional phenylalanine is given, (2) higher phenylalanine doses do lead to a further decrease of the occurrence of low phenylalanine concentrations however they increase tyrosine to concentrations above the target range and therefore may be less satisfactory and (3) the effect of phenylalanine supplementation, detection of low phenylalanine concentrations and possible phenylalanine deficiency should be analyzed using pre-midday meal blood samples which could be combined with an overnight fasted blood sample of the same day when in doubt. Further research should focus on if additional phenylalanine supplementation benefits long term growth and development. 

## Figures and Tables

**Table 1:** Overview of the different study periods with sample times.

		Period 1 Without Phenylalanine Supplementation		Period 2 First Phenylalanine Supplementation round (20 mg/kg/day)					Wash-out Period	Period 3 Second Phenylalanine Supplementation round (40 mg/kg/day)				
**Study day**		**1**	**2**	**3**	**4**	**5**	**6**	**7**	**8–14**	**15**	**16**	**17**	**18**	**19**
**Time blood spot**	**Break-fast**	X	X		X	X	X	X			X	X	X	X
	**Midday meal**	X	X		X	X	X	X			X	X	X	X
	**Evening meal**	X	X		X	X	X	X			X	X	X	X

**Table 2:** Patient characteristics.

Pat. Number	Age (year)	Gender	Weight (kg)	Standard Extra Phenyl-Alanine (mg/kg/day)	Natural Protein Intake (g/kg/day)	Total Protein Intake (g/kg/day)	NTBC Intake (mg/kg/day)	Phenylalanine Supplementation during Study	
								20 mg/kg Period	40 mg/kg Period
1	7.6	F	30	25.0	0.3	1.4	1.1	20.3	40.5
2	12.3	M	52	14.4	0.2	1.1	1.2	18.8	40.0
3	19.5	M	64		0.2	0.9	1.1	21.1	39.8
4	27.0	M	65	2.5	0.4	0.9	0.9	20.8	39.2
5	6.9	F	25	4.0	0.9	1.9	0.7	18.0	42.0
6	15.6	M	46		0.5	1.8	0.9	20.8	41.7
7	13.9	M	75		0.4	1.2	0.5	20.0	40.0
8	13.3	M	49		0.5	1.6	0.8	20.4	40.8
9	14.2	F	59		0.3	1.4	0.5	20.3	40.7
10	9.3	F	38		0.7	2.2	0.8	15.8	39.5
11	14.4	M	59		0.5	1.4	0.4	20.3	39.8

**Table 3:** Descriptive information about mean blood phenylalanine concentrations at different sample moments during the different study periods, presented with mean ± standard deviation (SD).

	Breakfast (µmol/L)	Midday Meal (µmol/L)	Evening Meal (µmol/L)
**Period 1 (0 mg/kg/day)**	50 ± 21	37 ± 15	43 ± 25
**Period 2 (20 mg/kg/day)**	51 ± 18	47 ± 19	48 ± 23
**Period 3 (40 mg/kg/day)**	52 ± 14	56 ± 20	58 ± 20

**Table 4:** Blood succinylacetone (SA) concentrations during the study.

	No Phenylalanine Supplementation	20 mg/kg Phenylalanine Supplementation	40 mg/kg Phenylalanine Supplementation
**SA < 0.1 µmol/L**	23 (66%)	41 (51%)	44 (57%)
**SA ≥ 0.1 µmol/L**	12 (34%)	39 (49%)	33 (43%)
**Total**	35 (100%)	80 (100%)	77 (100%)
